# Host-hijacking and planktonic piracy: how phages command the microbial high seas

**DOI:** 10.1186/s12985-019-1120-1

**Published:** 2019-02-01

**Authors:** Joanna Warwick-Dugdale, Holger H. Buchholz, Michael J. Allen, Ben Temperton

**Affiliations:** 1 Plymouth Marine Laboratory, Prospect Place, The Hoe, Plymouth, PL1 3DH UK; 2 University of Exeter, Geoffrey Pope Building, Stocker Road, Exeter, EX4 4QD UK

**Keywords:** AMGs, marine, Cyanophage, Nucleotide scavenging, Biogeochemical cycling, Host-virus interactions, Lysogeny

## Abstract

Microbial communities living in the oceans are major drivers of global biogeochemical cycles. With nutrients limited across vast swathes of the ocean, marine microbes eke out a living under constant assault from predatory viruses. Viral concentrations exceed those of their bacterial prey by an order of magnitude in surface water, making these obligate parasites the most abundant biological entities in the ocean. Like the pirates of the 17th and 18th centuries that hounded ships plying major trade and exploration routes, viruses have evolved mechanisms to hijack microbial cells and repurpose their cargo and indeed the vessels themselves to maximise viral propagation. Phenotypic reconfiguration of the host is often achieved through Auxiliary Metabolic Genes – genes originally derived from host genomes but maintained and adapted in viral genomes to redirect energy and substrates towards viral synthesis. In this review, we critically evaluate the literature describing the mechanisms used by bacteriophages to reconfigure host metabolism and to plunder intracellular resources to optimise viral production. We also highlight the mechanisms used when, in challenging environments, a ‘batten down the hatches’ strategy supersedes that of ‘plunder and pillage’. Here, the infecting virus increases host fitness through phenotypic augmentation in order to ride out the metaphorical storm, with a concomitant impact on host substrate uptake and metabolism, and ultimately, their interactions with their wider microbial community. Thus, the traditional view of the virus-host relationship as predator and prey does not fully characterise the variety or significance of the interactions observed. Recent advances in viral metagenomics have provided a tantalising glimpse of novel mechanisms of viral metabolic reprogramming in global oceans. Incorporation of these new findings into global biogeochemical models requires experimental evidence from model systems and major improvements in our ability to accurately predict protein function from sequence data.

## Background

Based on their extraordinary abundance and diversity, J.B.S. Haldane once quipped that ‘*The Creator would appear as endowed with a passion for stars, on the one hand, and for beetles on the other*’ [1]. In comparison, the Creator’s zeal for viruses would make stars and beetles appear to be a side-project performed with perfunctory indifference. It is estimated that there are a million viruses in the ocean for every star in the universe [2]. Assuming an average size of 100 nm in length, placed end-to-end marine viruses would stretch to our nearest neighbour star (Proxima Centauri, 4.22 light years away) and back. The vast majority of these viruses are obligate parasites of marine bacteria - the primary drivers of global carbon biogeochemistry [3]. With 10^28^ infections occurring per day in the oceans [4], these bacteriophages are responsible for up to 50% of bacterial mortality [5] and the daily release of 12.4 μg of organic carbon per litre of seawater, or an estimated 10 billion tons globally per day [5, 6]. Release of cellular substrates through lysis have been shown to stimulate surviving members of the community and increase microbial productivity through nutrient recycling [7–9]. Thus, marine viruses are a major component of global carbon cycling.

Lysis and release of cellular material as dissolved organic matter is only the final step of a complex host-virus interaction where the invading pathogen can manipulate host metabolism and alter its phenotype to favour viral replication at the expense of host function. Once a virus has overcome host defences, lytic viral infection typically involves a shut-down of host metabolism, followed by degradation of macromolecules and scavenging of intracellular resources. Virally encoded ‘Auxiliary Metabolic Genes’ (AMGs) are expressed during infection to augment and redirect energy and resources towards viral production [10–13]. These AMGs are often repurposed versions of host-genes picked up during ancestral infections, evolving separately to improve viral fitness [14, 15]. Conservation of AMGs across phage lineages suggests that not only are such genes critical for viral success, but that viral modulation of marine nutrient cycling via metabolic hijacking is an important, but understudied component of biogeochemical cycles. Furthermore, viral replication through lysis is only beneficial to the virus if upon release, its progeny can infect other susceptible cells. This process is governed by host availability and density-dependent selection. In resource-limited or otherwise challenging environments viral fitness may be better served by maximising host fitness until such time as the conditions are met for a lytic approach to be favourable. Single amplified genomes of bacterial cells from marine environments have shown that ~ 1/3 contain viral sequences [16, 17]. Between 28 and 71% of marine bacterial isolates contain inducible prophage, with greater occurrence of lysogeny associated with low-nutrient environments [18–21]. In extreme environments such as hot-springs, nearly all cells contained a viral signature [22]. In lysogenic cells, the viruses are not passive passengers, but actively promote the host fitness through expression of viral genes increasing metabolic flexibility, enabling antibiotic resistance, toxin production and immunity to similar viruses [23] (reviewed in [24]). The aim of this review is to illustrate the different ways in which bacteriophages alter the function of their hosts, emphasising how marine viruses modify the metabolism of environmentally relevant bacteria and in turn their impact on global carbon biogeochemistry. We also discuss how novel molecular methods are providing greater insight into the breadth and scale of viral hijacking in the global ocean.

## Plunder and pillage - redirecting cellular metabolism to maximise viral production

**Fig. 1:**
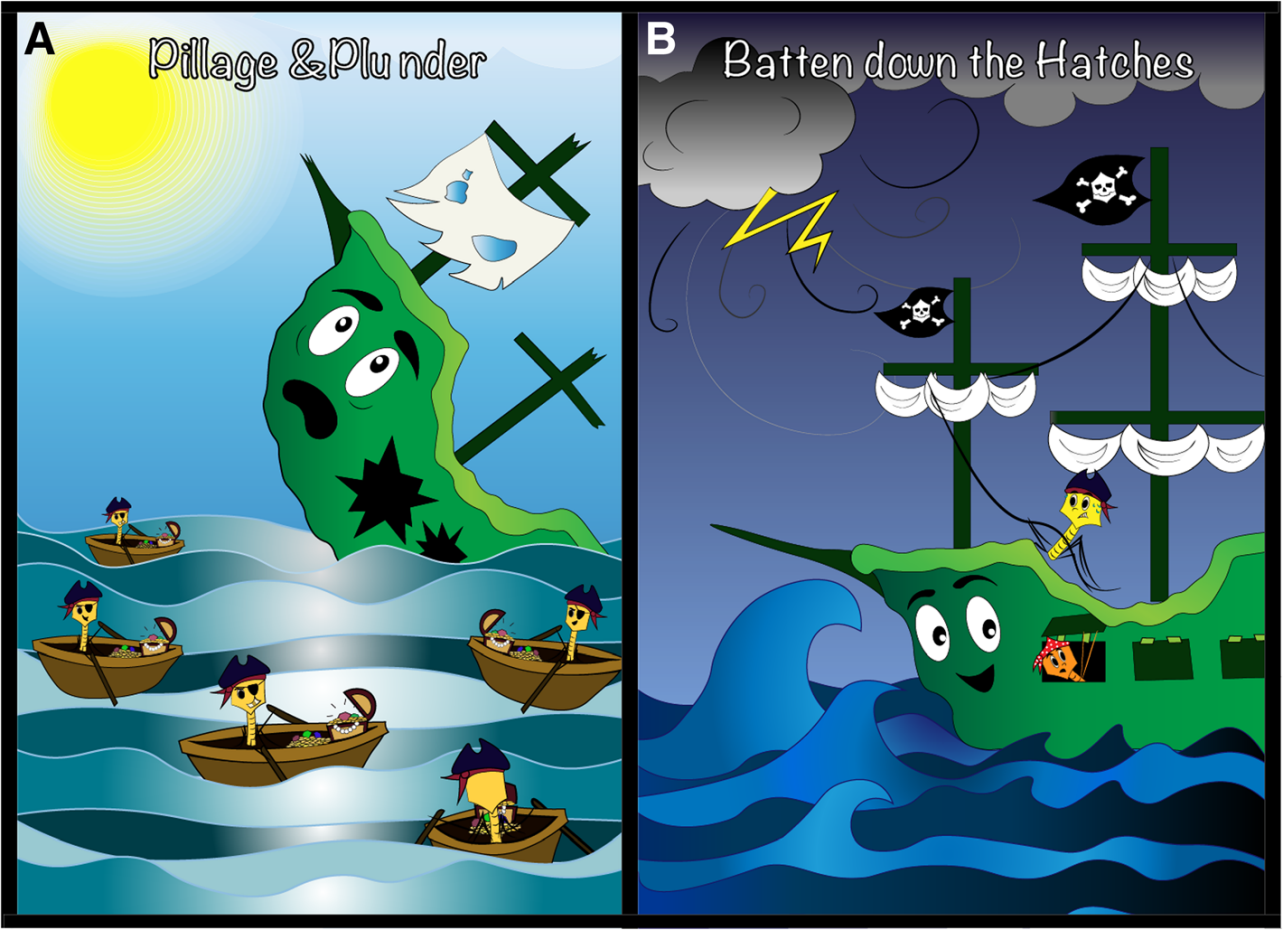
A cartoonist’s depiction of the two types of host-virus interactions in the oceans. Under the ‘Pillage and Plunder’ model (**a**), the virus infects its host and redirects energy and substrates towards viral replication before lysing the cell and releasing viral progeny for further infections. Under the ‘Batten down the hatches’ model (**b**), viral fitness is improved by increasing host fitness, either by augmenting metabolic flexibility through virally-encoded genes, increasing resistance against other viruses, or by curbing host metabolism to maximise host survival under nutrient limitation

First, we will consider the classic view of viral predation, where the virus replicates by lytic infection and redirects host resources for the purpose of viral production that ends in cell lysis and release of viral progeny (the ‘Plunder and Pillage’ strategy, Fig. 1a). Once a virus docks with a susceptible cell and successfully injects its genome and associated proteins into the host, hijacking of host metabolism is swift and efficient. Infection in marine T4-like cyanophages follows a similar pattern to that observed in T4 infections in *Escherichia coli*, with 3 distinct phases of transcription of viral genes: (1) defence neutralisation and host metabolic rewiring; (2) synthesis of viral components; and (3) viral assembly [25]. These stages are referred to as ‘early’, ‘middle’ and ‘late’ stages, respectively. Examples of expression of early stage genes include the infection of the marine Bacteroidetes *Cellulophaga baltica*. Infection with a specialist phage was initiated with expression of 15 genes putatively involved in overcoming host defences. Of these, one was an anti-restriction defence gene thought to provide viral defence against host degradation of viral DNA. The remaining 14 genes had unknown function but shared a promoter with the anti-restriction defence and were located in the same genomic neighbourhood, inferring a complementary functional role [13]. A study of the transcriptional response to infection by the cyanophage Syn9 in multiple *Synechococcus* hosts showed that within the first 30 min following infection, almost all transcription is associated with phage encoded genes and within the first hour of infection ~ 80% of cellular DNA has been scavenged for resources [25]. Reconfiguration of cellular transcription machinery is often achieved either by altering host RNA polymerases to favour viral promoters using phage factors packaged within the viral capsid and delivered during infection, or by direct delivery of a virally-encoded RNA polymerase [25–28].

Once the cellular machinery has been repurposed for viral production, the ‘middle’ stage genes are expressed, which include mechanisms to manipulate host metabolism. Termed “Auxiliary Metabolic Genes” (AMGs), many of these viral genes encode for central metabolic proteins and are conserved across different marine phage lineages, signifying their importance to viral success [29]. Two different classes of AMG have been described (reviewed in [30]): to be categorised as Class I, a gene must encode a protein with a central metabolic function, for example in photosynthesis, carbohydrate metabolism or amino acid metabolism, and it will appear in a Kyoto Encyclopaedia of Genes and Genomes (KEGG) metabolic pathway [31]; Class II AMGs have been defined as those which encode proteins that have only general, undefined metabolic roles, or peripheral roles (examples include assembly or membrane transport functions) and therefore do not appear in KEGG metabolic pathways [30, 32]. Table 1 provides numerous examples of metabolic pathways manipulated by virally-encoded genes in marine and non-marine systems, as identified from both experimental and metagenomic analyses.

**Table 1:** Examples of studies that reported/predicted phage-mediated alteration of metabolic function in prokaryotic hosts

Host/s	Phage/s; cycle (if known)	Modification/Phenomena (molecular; physiological; phenotypic)	Observed effect (O) or Predicted effect (P) on host metabolism/ host survival	References
*Vibrio cholera*	VPIΦ and CTXΦ; Lysogenic	Insertion of VPIΦ results in toxin-coregulated pilus (TCP) expression; TCP-facilitated CTXIΦ insertion into host genome	(O) Expression of cholera toxin	[67, 68]
*Escherichia coli*	933 W; Lysogenic to lytic switch	Induction of 933 W prophages that encode for both shiga toxin (Stx) and a cleavable repressor	(O) Greatly increases *stx* gene expression, and therefore bacterial production and release of Stx.	[65]
*Staphylococcus aureus*	Φ13; Lysogenic	Integration of Φ13 genome with beta-toxin gene (*hlb*)	(O) Loss of beta-toxin expression (Note: beta-toxin is a sphingomyelinase)	[71]
*Escherichia coli*	λ; Lysogenic	λ cI protein expression; cI binds to *pckA* regulatory region preventing transcription	(O) Suppression of phosphoenolpyruvate carboxykinase production & gluconeogenesis; reduced growth rate; predation avoidance	[66]
*Vibrio harveyi* 645; 20; 45	VHML; Lysogenic	Integration of VHML genome via transposition	(O) Broad suppression of substrate utilization; changes in d-gluconate utilization (625); c-glutamyl transpeptidase activity (20); and sulfatase activity (45)	[69, 70]
*Listonella pelagia*	ΦHSIC; Pseudolysogenic	Chromosomal integration of prophage	(O) Reduction in substrate utilization	[21, 110]
*Cellulophaga baltica MM#3*	ΦS_M_ and ΦS_T_; Lytic	On evolution of phage resistance: possible adaptation of amino acid transporters (likely phage receptors) in cell membrane	(O) Reduction in ability to metabolise various carbon sources, including many amino acids	[111]
*Synechococcus WH8109*	Cyanophage Syn9; lytic	Phage encoded carbon metabolism genes cp12, *talC, psbA, zwf, gnd*, and *nrdA/nrdB*, co-expressed in early infection; two-fold increase in NADPH/NADP ratio	(P) ‘light reactions’ decoupled from ‘dark reactions; ATP & NADPH directed away from the Calvin Cycle. likely towards phage dNTP biosynthesis	[10]
*Synechococcus WH8017*	S-SM2; lytic	Phage encodes genes for photosynthesis (PSII): *psbA*; *psbD*, and carbon metabolism genes: *gnd*; *tal; zwf;* CP12	(P) Photosynthesis augmented during infection; carbon redirected from glucose and amino acid production to ribose-5P and NAPDH generation (for dNTP synthesis), via PPP-mediated glucose reduction	[42, 53]
*Cyanobacteria: various Prochlorococcus and Synechococcus strains*	Various: 42 cultured cyanophages	88% of cyanophage genomes include *psbA*; 50% code for both *psbA* and *psbD* (PSII genes)	(P) Boost to phototrophic metabolism during infection.	[33]
*Cyanobacteria*	Un-cultured cyanophages	Phage-encoded photosystem I genes *psa*A, B, C, D, E, K and JF (from environmental samples)	(P) Channelling of reducing power from respiratory chain towards PSI, possibly for ATP generation	[34]
*Prochlorococcus MIT9515*	P-TIM68; lytic	Phage encoded photosystem I and II proteins incorporated into host membrane	(O) Photosynthetic capacity maintained; enhanced cyclic electron flow around PSI; (P) Generation additional ATP for phage replication	[37]
*Vibrios (including V. parahaemolyticus*	KVP40; lytic	Phage ORFs code for: PhoH; putative pyridine nucleotide (NAD^+^) salvage pathway, and hydrolysis of NADH	(P) Facilitates cross-membrane transport of NAD^+^ precursors, NAD^+^ synthesis, and cycling of NADH back to precursors.	[112]
*Various (marine metagenomic Assemblages)*	Various (marine viral metagenomes)	Most abundant putative viral-encoded enzymes: riboreductases; carboxylyases and transferases; *psbA* genes.	(P) Aids scavenging of host nucleotides (e.g. Riboreductases); supports host metabolism during the infection cycle (e.g., carboxylyases; transferases and D1 protein)	[113]
*Various: from 22 ‘ultra-clean’ viromes in POV dataset*	Various; classified via protein cluster (PC) generation	35 carbon pathway AMGs, representing a near-full central carbon metabolism gene complement.	(P) In oligotrophic environments, AMGs may redirect host carbon flux into energy production and the replication of viral DNA.	[53]
*Various: from 32 viromes in POV dataset*	Various; classified via PC generation	32 new viral AMGs (9 core; 20 photic; 3 aphotic): 9 encode Fe-S cluster proteins and genes associated with DNA replication initiation (*DnaA*), DNA repair (*dut*; *radA*) and motility augmentation (*psel*).	(P) Fe-S cluster modulation may drive phage production (in the photic zone); Genes associated with DNA replication and repair, and motility augmentation could assist high-pressure deep-sea survival.	[30]
*Various: 127 SAGs from uncultivated SUP05 bacteria*	Various: 69 viral contigs (from SUP05 SAGs)	4 putative AMGs (encoded by 12 viral contigs): *phoH* (on a bona fide viral contig); 2OG; 2OG-FeII oxygenase, *tctA* (protein domain only); and *dsrC.*	(P) *dsrC* likely functional in SUP05 sulfur cycling; characterisation of viral DsrC needed to elucidate roles of viruses in modulating electron transfer during viral infection.	[16]
*Various: Actinobacteria, proteobacteria (α; δ; γ) Bacteroidetes, Cyanobacteria, Deferribacteres*	Various, inc. members of T4 (superfamily) and T7 (genus)	243 putative AMGs (95 previously known [6]) including *dsr*C (11 genes), *sox*YZ (4 genes), both originating from T4 superfamily; *P-II* (encodes a nitrogen metabolism regulator) and *amo*C (encodes ammonia monooxygenase sub-unit)	(P) Viral roles in: Sulfur oxidation, via Dsr and Sox pathways; Nitrogen cycling (influenced by *P-II*), with potential for alternative pathways of N and NH_3_ uptake during N starvation, and NH_3_oxidation (via *amo*C).	[47]
*Various: 113 genomes (marine bacteria)*	Various: 64 pro-phage-like elements (21 GTAs)	High relative incidence of transcriptional regulatory and repressor-like proteins in putative prophages (comparison: lytic phages)	(P) Suppresses non-essential host metabolic activities in unfavourable environments/periods	[21]
*Listeria monocytogenes*	‘A118-like prophage’ (reversible excision)	*comK* gene, encoding *L. monocytogenes* competence system master regulator, is activated by the excision of A118-like prophage	(O) A118-like prophage is excised only when a *L. monocytogenes* cell is engulfed by a phagosome: the host’s activated competence system facilitates escape, after which prophage reintegrate with host *comK* gene, deactivating host’s competence system	[74]
*Anabaena spp.; Nostoc spp.*	Non-infective ‘prophages’ (x 3; non-reversible excision)	Recombinases (prophage-encoded) act to excise prophages from 3 host genes that are involved in nitrogen fixation (*nifD*; *fdxN*; *hupL*)	(O) In low nitrogen environments, excision of prophages from host N-fixation genes enables conversion of host cell to form nitrogen-fixing heterocysts	[74]
*Synechococcus elongatus*	Cyanophage AS-1	Prevents normal ppGpp accumulation under nutrient limitation, and the corresponding expression of genes for starvation survival	(O) Inhibits the host’s natural starvation response under nutrient limitation; (P) promotes metabolic activity otherwise undertaken only when food is plentiful, facilitating phage production in low nutrient conditions	[14, 114]

Abbreviations not used in the main text: *ORF* Open Reading Frame, *dut* Deoxyuridine triphophatase, *radA* DNA recombination protein, *pseI* Pseudaminic synthase, *2OG* 2-oxoglutarate; *2OG-FeII oxygenase* Fe (II)-dependent oxygenase superfamily, *tctA* Tripartite tricarboxylate transporter, *GTA* Gene Transfer Agents

Virus-directed augmentation and redirection of host energy and resources to increase viral progeny production has been observed in both marine heterotrophs and phototrophs, with the latter the focus of much of the seminal research in this area. The lytic cyanophage Syn9 encodes photosystem II core protein D1 (which enables transfer of electrons from water to plastoquinone), as well as genes catalysing the synthesis of deoxyribonucleotides (*nrdA/nrdB*) using NADPH as a terminal electron donor [10]. Indeed, viral encoding of genes associated with photosystem II is common in cyanophages, with 88% of cyanophage genomes containing *psbA* and 50% containing both *psbA* and *psbD* [33]. Phages encoding genes associated with photosystem I were identified in metagenomic data and postulated to increase ATP concentrations to power viral production by promoting cyclic photophosphorylation, at the expense of reducing power for CO_2_ fixation [34]. Interestingly, in silico modelling of the advantage conferred by a phage encoding a *psbA* gene suggested that the increase in energy available to the virus for replication is negated by the increased cost of encoding an extra gene. However, under high-light conditions, the virus gains an advantage by replacing photosystem II machinery damaged by light stress [35, 36]. Thus, the presence of photosystem genes on viral genomes is a function of host ecotypic distribution and irradiance sensitivity. Recently a novel *Prochlorococcus*-infecting cyanophage (P-TIM68) was isolated that encodes viral genes for photosystem II as well as a cassette for photosystem I (*psaABCDEFJK*) - the first known example of a phage to manipulate both photosystems during infection. Predictions of enhanced cyclic electron flow around photosystem I were also confirmed [37]. Typically, these genes are co-expressed during early and middle infection and result in a decoupling of photosynthetic ‘light reactions’ from ‘dark reactions’, directing ATP and reducing power away from CO_2_ fixation and towards nucleotide biosynthesis for phage replication [10, 37].

Re-routing of photosynthesis-derived energy to increase viral production is just one of many strategies viruses employ during infection to plunder the cellular resources of their hosts. The primary limiting nutrients in marine environments are phosphate (P), nitrate (N) and iron (Fe) [38–40]. Therefore it is perhaps unsurprising that marine viruses encode AMGs to augment host uptake of these resources during infection. Genomes of viruses isolated from P-limited environments have been found to contain more AMGs associated with P-uptake than those from P-replete environments [41]. T4-like cyanophages, S-SM1 and S-SM2, encode an alkaline phosphatase, putatively enabling cleavage of phosphate from organic phosphate sources [42]. Under P-limitation, hosts increase production of the phosphate transporter PstS to maximise uptake, and in P-limited marine environments PstS is one of the most abundant proteins identified in metaproteomic datasets [43]. Both virally-encoded *pstS* and *phoA* were over-represented in viral genomes from the North Atlantic Subtropical Gyre, and occurred at similar frequencies to signature core genes. In the North Pacific Subtropical Gyre, *phoA* was much less abundant in viral genomes, providing evidence of niche-separation of AMGs corresponding to limiting nutrients [41]. Using model cyanophage/host systems, Zeng and Chisholm showed that both virally-encoded *pstS* and *phoA* AMGs are upregulated during infection of P-limited hosts [15]. The phosphate-regulon associated gene, *phoH*, is so common in viral genomes it has been proposed as a signature gene for measuring phage diversity [44]. However, it is worth noting that the function of virally-encoded *phoH* is not yet clear, and *phoH* expression in phosphate limited conditions appears to vary between hosts [45, 46].

Viral manipulation of cellular nitrogen and sulfur levels during infection is perhaps less well documented in cultured host-virus systems than phosphate regulation, but recent research is providing evidence that it is no less important. Phage genomes have been found to encode numerous proteins to manipulate concentrations of 2-oxoglutarate, which in turn, regulates cellular N-limitation response via the promotor NtcA [42]. Beyond cultured representatives, a recent global study of viral metagenomics identified 243 putative viral AMGs including genes for photosystem II, ammonium transporters (*amt*) and ammonia monooxygenases (*amoC*), as well as genes associated with sulfur reduction (*dsr*) and oxidation (*sox*) [47]. Screening of marine cellular metagenomes from the Tara Oceans cruises revealed that not only do *amoC* genes encoded by viruses infecting Thaumarchaeota form a distinct phylogenetic clade, they can also comprise up to half of the total abundance of *amoC* in some metagenomic datasets [48]. Nitrate reductase genes (*nar*) have also been found in viromes from deep-sea hydrothermal vents [49]. Evidence of virally-encoded components of dissimilatory sulfite reductase complex (Rdsr) have been found in viruses infecting the uncultivated chemoautotrophic gammaproteobacterial clade SUP05 [16, 50, 51]. SUP05 are important drivers of sulfur cycling [52] and peaks in abundance of virally-encoded *dsrC* were observed during SUP05 blooms. Investigators concluded that phage-encoded DsrC is likely to function in the sulfur cycling of infected SUP05, and may modulate vital electron transfer reactions. Evidence indicating increased viral infection of SUP05 with increasing depth, and decreasing O_2_ levels, underlines the potential significance of this finding [16].

Virally-encoded genes associated with carbon uptake have also been identified in both model experimental systems and viral metagenomes, including those involved in uptake and metabolism of amino acids (*speD*, *cysK/M*, *metK*, *dapC*) and carbohydrates (*manA*, *rpiB*, *glgA*) [30, 53]. Cyanophage genomes include genes (*talC*, *zwf*, *gnd*) to divert carbon towards the pentose phosphate pathway by converting glyceraldehyde-3P to fructose-6P, which is subsequently converted to reducing power and the synthesis of dNTPs for phage replication [10]. Viral metagenomics also identified other virally encoded genes involved in glycolysis (*manA*) and a glycogen synthase (*glgA*), with the latter identified in all examined viral metagenomes [53]. In this work, Hurwitz and colleagues postulated *glgA* was used to trigger a starvation response in the host in order to push carbon through non-glycolytic pathways that promote dNTP biosynthesis. Manipulation of succinate through the glyoxylate shunt is thought to facilitate energy production at the expense of amino acid synthesis, particularly in nutrient limited, deep ocean samples. Intracellular carbon is also redirected during viral infection for energy via virally-encoded genes for the Entner-Doudoroff pathway, the TCA cycle and fatty acid metabolism [53].

So how does expression of viral AMGs during infection influence cells at the fundamental level of metabolite synthesis and utilisation? The influence of viral hijacking at the metabolite level lacks a representative study in marine systems, but De Smet and colleagues performed an exemplary study in *Pseudomonas aeruginosa* PAO1 and compared responses across host-virus infections between one host and six different phages [12]. Following infection, concentration of 92 out 375 detectable host metabolites were significantly altered, showing a major rewiring of host metabolism. Only 9 of these metabolites, all associated with nucleotide metabolism, were altered by all six tested phages. This indicates that beyond nucleotide synthesis for viral replication, the impact of viral hijacking on host metabolism is not conserved even within a single host species. Larger phage genomes encoded a larger suite of metabolic machinery for viral production, whereas smaller genomes relied more on host machinery and scavenging of resources. Phages which encoded one or more RNA polymerase shut down host transcription, and initiated their own transcription machinery. Phages with smaller genomes rewired host RNA polymerases directly to increase promoter specificity towards phage-specific promoters. Interestingly, hijacking of host metabolism appeared to favour pyrimidine production, with purine synthesis enriched only in phage YuA. Infection with YuA (genome size of 58.6 kb) also resulted in major depletion of cellular resources, recycling them for phage synthesis. In comparison, phage phiKZ (which has a 280kbp genome encoding 306 open reading frames) had almost no impact on host metabolite concentrations [12].

Efficiency in host hijacking is thus not universal, and tends to be higher in infections between specialist phages and their preferred host (which are the focus of many host-phage model systems). Generalist phages that infect multiple hosts tend to have less efficient infections, fail to completely suppress host translation and transcription, have longer latent periods and decoupled translation and transcription [12, 13]. The ‘plunder and pillage’ strategy was not observed in a generalist phage infection of *Cellulophaga baltica* [13], and it is possible that utilisation of AMGs for hijacking of host metabolism is not a feature of generalist infections. Yet, members of the abundant marine Myoviridae infecting *Synechococcus* and *Prochlorococcus* include many generalists, some capable of infecting both genera [54, 55]. The fitness advantage to adopting a broad host range, at the expense of efficient infection makes ecological sense when one considers that temporal patterns in marine environments often result in transition from *Synechococcus*-dominated to *Prochlorococcus*-dominated communities as nutrient availability waxes and wanes over seasonal, depth and diel gradients [56–58]. Furthermore, recent evidence from an experimental evolution experiment suggests that infection of sub-optimal hosts increases mutation rates and diversification of phage populations compared to those infecting optimal hosts [59].

## Batten down the hatches: Increased viral fitness through increased host fitness

From the perspective of viral fitness, the benefit of driving host energy and metabolites towards viral production during lytic infection only confers an advantage if viral progeny released following a lytic event can successfully infect new, actively growing hosts. Imagine a band of naïve pirates in the doldrums of a seldom-travelled ocean gyre. They seize a ship, kill the crew and strip its cargo with alarming efficiency, before scuppering the vessel and sailing off in rowboats. With no land in sight, nor new vessel to capture, their career as pirates would be short and they would soon succumb to the tropical sun, deprived of life-giving resources. In contrast, a savvy band of pirates would instead remain aboard the seized vessel, secure the mainmast during storms that may arise, ration the rum and steer her towards more profitable waters. Many marine viruses display a similar ‘batten down the hatches’ strategy by foregoing a lytic life cycle and instead increasing the fitness of the infected cell phenotype through metabolic manipulation (Fig. 1b). Here, instead of producing viral progeny, the infecting virus is maintained either by integration into the host genome (prophage) or as an extrachromosomal element to convert the host into a lysogen. Lysogeny includes a broad range of mechanisms including chronic infection (slow release of viral particles without killing the host), pseudolysogeny (simultaneous high viral production without host lysis) and polylysogeny (infection of multiple viruses within the same host) (reviewed in [21]). Numerous studies of the prevalence of lysogeny in marine systems all suggest that lysogeny is favoured in low productivity environments [21, 60, 61]. Chemical induction of lytic viral production in natural marine communities using mitomycin C showed the switch only occurred at cellular concentrations > 10^6^ cells per mL [62]. A significant decrease in viral abundance has been observed at depth, corresponding to host abundance an order of magnitude lower than those at the surface [63]. These lower virus-to-microbe ratios are often interpreted as evidence of increased lysogeny. A recent meta-analysis of virus-to-microbe ratios across global oceans showed that this ratio varies from 1.4 to 160 and suggested that host-virus ratios are shaped by complex feedback mechanisms between nutrient availability, evolutionary history and selection pressures [64].

Regardless of the mechanism used to maintain the virus within the host during lysogeny, the effect of phage on host phenotype has been extensively studied in important pathogens such as *E. coli* [65, 66], *Vibrio cholerae* [67, 68], *Vibrio harveyi* [69, 70]*, Staphylococcus aureus* [71] and *Listeria monocytogenes* [72] (reviewed in [73, 74]) (Table 1). However, relatively few studies into lysogeny in marine host-virus model systems have been performed. When Yu and colleagues isolated a *Pseudoalteromonas* strain from Arctic sea ice containing a filamentous phage, they noticed it had lower growth rates and cell density, and lower tolerance of NaCl and reactive oxygen species than when it was cured of the infection. Transcriptional analysis showed downregulation of succinyl-CoA synthetase and succinate dehydrogenase indicating virally-mediated suppression of central carbon metabolism. However, presence of the phage increased host motility. They postulated that the presence of the phage increased host fitness during the nutrient-limited polar winters by slowing down host metabolism and increasing its capacity to find new nutrient sources in the heterogeneous structure of the sea ice in which it lived [75]. Using metagenomic analyses, Brum and colleagues postulated that a similar mechanism increased host fitness in Antarctic bacterial communities during times of low nutrients and explained an observed seasonal prevalence of lysogeny prior to the summer blooms [76]. The underpinning mechanisms are possibly similar to those observed in the *E. coli* phage λ. This lysogen maintains integration in the host genome via a phage-encoded repressor known as *cI*. *cI* also represses the host gene *pckA,* which encodes a protein for the conversion of oxaloacetate to phosphoenolpyruvate. Thus, lysogeny results in a decoupling of central carbon metabolism from cellular synthesis, reducing host growth rate and potentially conferring a selective advantage in hosts within nutrient-poor environments [66]. Integration of the viral genome into the host genome can interrupt metabolic genes and effectively act as regulatory mechanisms, either at the level of the individual cell by repeated integration and excision, or at the population level by non-reversible lysogeny suppressing genes in a subpopulation [74]. Although there are no known examples of the former in marine bacteria, viruses infecting the cyanobacteria *Anabena* spp. and *Nostoc* spp. appear to regulate nitrogen-fixation through active lysogeny. Here, prophages interrupting N-fixation genes (*nifD*, *fdxN* and *hupL*) are excised from the genome during N-limitation, re-activating the genes (reviewed in [74]). Many metabolic genes identified in viral metagenomes are predicted to confer a selective advantage to hosts including sulfur oxidation genes [16, 50] and genes associated with adaptation to high-pressures associated with depth [30]. One study of viral metagenomes from hydrothermal vents identified virally-encoded genes associated with pyrimidine, alanine, aspartate, glutamine, nitrogen, amino and nucleotide sugar metabolism. Pathway analysis suggested that viral AMGs allowed for branched metabolic pathways to alternative products that would provide additional metabolic flexibility to the host, thus increasing host fitness [49].

## Metabolic false flags

In March 1723, the pirate Captain Low approached a Spanish merchant ship in the Bay of Honduras under the Spanish colors. Once they drew near the vessel, they:

‘*hauled them down, hoisted up their black flag, fired a broadside and boarded her*’ [77].

Indeed, it was commonplace for pirates to sail under false flags of different countries in order to prevent their targets from identifying them as a threat until it was too late. Similarly, viruses encode genetic tools to prevent a hijacked host from recognising an infection and taking appropriate action. As internal concentrations of cellular substrates decrease, cells can undergo a ‘stringent response’ where they down-regulate growth functions and, in some cases, initiate programmed cell death, regulated by the toxin-antitoxin pair MazEF. Nutrient limitation results in uncharged tRNAs, which triggers increasing concentrations of guanosine tetraphosphate (ppGpp). This in turn inhibits RNA transcription and promotes transcription of the global stress response regulator RpoS, shifting the cell into stationary phase [78]. Increasing concentrations of ppGpp also increases production of MazF, a toxin that inhibits protein synthesis (resulting in cell dormancy). In some circumstances, MazF also initiates programmable cell death, with different mechanisms used under different stressors such as antibiotic treatment or DNA damage [79, 80]. Programmable cell death is thought to sacrifice a large proportion of a population, so that a small sub-population can survive by recycling released nutrients [80]. Programmable cell death regulated by MazEF has been shown to play a role in the survival of a bacterial population against phage infection*.* Hazan and Engelberg-Kulka showed that 400 times more viral progeny were produced in Δ*mazEF E. coli* cells infected with phage P1 compared to wild-type cells. In addition, when lysogens were mixed with non-lysogens, the Δ*mazEF* cells were susceptible to infection and lysed, whereas the non-lysogen wild-type cells were not infected by the induced phage. They suggested that it is likely the wild-type lysogens were killed by MazEF, preventing viral replication to the benefit of the population [81]. Similar observations of the role of toxin-antitoxin mechanisms for phage resistance were observed in *Erwinia carotovora*, where expression of the toxin-antitoxin system conferred resistance to a broad range of viruses [82].

Given its dual role as both a regulator of the stringent response and of MazEF-regulated resistance and cell death, increasing cellular levels of ppGpp pose a clear threat to viral replication. Many viruses are dependent on host RNA transcription for replication, and viral replication has been shown to be drastically reduced or suspended in infected *E. coli* cells that enter stationary phase [83]. Cell death limits the spread of phages through a population by reducing host density and purges lysogens from the population. Thus, is of little surprise that in the arms-race between viruses and their hosts, the viruses have commandeered a ppGpp regulator to counter host defence mechanisms. MazG has been shown to decrease cellular concentrations of ppGpp in *E. coli* and thus repress production of MazF [79]. Thus in the host MazG serves as a switch to recover from a starvation response upon return to nutrient replete conditions and to abort programmable cell death. *mazG* is common in viral genomes from marine environments and has been reported in viruses infecting both heterotrophs [84, 85] and phototrophs [14, 86]. The oligotrophic nature of vast areas of the ocean make it likely that a starvation state for host cells is the norm, resulting in high cellular concentrations of ppGpp and minimal transcription. It is likely that phages overcome this limitation by using virally-encoded MazG to deplete cellular ppGpp concentrations and thus force the infected cell to respond as if it were nutrient-replete, enabling transcription so that it may be hijacked for viral replication [87]. In addition, suppression of programmable cell death may prevent the host population from sacrificing itself in order to limit viral infection. Recent work in has shown that phages can utilise host quorum sensing to coordinate a lytic-lysogenic switch using a phage-derived oligopeptide signal [88] . Given that programmable cell death has been shown to be orchestrated through quorum sensing [89], and that suppression of *mazEF* expression increases host resistance to phages, it is conceivable that infecting viruses can use MazG to manipulate ppGpp concentrations and thus maintain the susceptibility of hosts within a population to subsequent infection by viral progeny. In pirate terms, viruses have evolved the capacity to sail under a false flag, disabling host alarm systems until it is too late. Further experimental evidence of the use of MazG and its effects on marine populations is required to explore whether this phenomenon is observed in nature.

## Future understanding of host-virus interactions

The current understanding of how viruses hijack host metabolism during infection is the result of both culturing experiments in model systems; and culture-independent techniques such as viral metagenomics. Culture-dependant techniques have significant advantages: Model systems enable us to study the relationship between host and viruses in controlled conditions; viral replication cycles and critical parameters (e.g. host range; burst size) can be defined, and the functionality of viral genes may be determined in vitro. In culture, predator-prey interactions can be isolated from those occurring in complex microbial communities. This reduction in complexity allows for investigation into how transcriptomes, proteomes and metabolites are altered during infection and thus enable a systems-biology approach to understanding complex metabolic cascades and regulation (e.g. [12, 13, 25, 45, 90]. However, many important marine taxa have, to date, resisted efforts to culture them [91]. Consequently, our model systems of host-virus interactions in marine systems are limited to a handful of taxa, with a major focus on the cyanobacteria and a limited number of heterotrophic hosts.

For systems outside of cultured representatives, viral metagenomic studies to date have provided major insights into to viral taxonomic and functional distribution and diversity [30, 32, 47, 53]. Understanding the extent and mechanisms of metabolic hijacking by marine viruses using metagenomic data comes with its own challenges: Firstly, in any viral metagenome there is the possibility of contaminating cellular DNA or randomly packaged host DNA encapsulated in gene transfer agent particles [32, 92]. As viral genomes are assembled from short read data, there is the possibility of cellular functions being misassembled into viral contigs. In such circumstances, the function may be interpreted as a novel AMG acquired by the virus to improve fitness, rather than as an artefact of bioinformatic processing [93, 94], with a concomitant over-estimation in the degree of viral piracy that occurs in marine systems. Secondly, some of the most cosmopolitan and dominant viruses on Earth are challenging to assemble using short-read technology and are under-represented in marine viral metagenomes [95, 96]. Complementary approaches to construct viral genomes from environmental samples such as single cell genomics and the development of long-read viral sequencing can alleviate these problems to some degree. Assembly of short-read data from a genome amplified from a single cell or single virus cell vastly reduces the complexity of the De Bruijn Graph and captures taxa missing from shotgun metagenomic approaches.

This approach has successfully to identify new viruses and novel AMGs [16, 17, 96]. Long-read viral metagenomics [97] offers the potential to accurately identify putative AMGs as viral, rather than cellular contaminants, by capturing the gene neighbourhood of the AMG to reliably assess its viral origins. Capturing full length viral genomes on single reads is now technically feasible and will provide a powerful tool to explore how AMGs are distributed within viral populations and how their evolution is impacted by recombination, shown to be the dominant form of mutation in some phages [98]. Long-read metagenomics from cellular fractions will better quantify the extent of lysogeny within a population by capturing integrated viral genomes on single reads.

It is worth noting however, that no matter how sophisticated sequencing methods become, perhaps the greatest barrier to understanding how marine viruses influence cellular metabolism during infection lies in our extremely limited capacity to identify the function of viral genes, in both cultured isolates and genomes constructed from environmental DNA. Whilst machine learning approaches are rising to meet this challenge [32, 99], one must consider that the scale of the ‘known unknowns’ is vast, with 63–93% of protein sequence space lacking functional or taxonomic annotations [100]. < 1% of viral populations in the Pacific Ocean Virome had a closely related taxonomic representative in culture [101]. Methods to identify viral host range through computational methods such as correlative abundance [102] or nucleotide composition [103, 104], are undergoing rapid development, but must be used cautiously for inferring ecological patterns [105]. Despite these challenges, the last decade has seen a dramatic improvement in our capacity to generate and interpret viral metagenomic data, largely driven by efforts to understand marine systems (e.g. [47]). This improvement has revealed a growing body of evidence identifying viruses as important agents in global carbon biogeochemical cycles, through: 1) lysis-dependent nutrient cycling and increased community productivity [7–9]; 2) influences on host-substrate interactions through auxiliary metabolic genes, shaped through viral evolution (reviewed in [11]). Viral metagenomics allows microbial ecologists to directly ascribe such functions to viruses and provides relative quantitation of viral populations and genetic diversity in a way that is challenging from cellular metagenomic data. More recently, these methods have been applied to medical microbiology and have similarly established viruses as an equally important component of the human microbiome alongside their cellular counterparts (reviewed in [106]). Indeed, there is a growing consensus that our view of microbial ecology must put viruses centre stage as key players in shaping community structure and function. Increasing interest in the role of viruses in microbiomes will undoubtedly catalyse a feedback loop that energises the development of novel bioinformatic and culturing methods. Such tools will ultimately overcome the technical limitations previously outlined, revealing more of the metabolic capacity for cellular piracy encoded within viral sequence space.

## Conclusion

The contemporary image of pirates is typically one of swashbuckling, romantic characters of Robert Louis Stevenson’s *Treasure Island* and J.M. Barrie’s *Peter Pan*. Piecemeal accounts and a lack of historical records has enabled myth and legend to supersede the violent and unpleasant reality written in Charles Johnson’s *A General History of the Robberies and Murders of the Most Notorious Pyrates* [107]. Similarly, the relationships between marine viruses and their hosts have been considered through the paradigm of predator and prey, with much research focused on viruses as agents of top-down control. Our understanding of viral impact on host metabolism in marine systems is derived from a few model systems, or inferred from model systems of medically relevant pathogens that have evolved in nutrient-rich environments supporting high cellular densities. It is now clear however that lysogeny is common in marine systems and has the capacity for reconfiguration of host metabolism and increasing host fitness during frequent periods of nutrient limitation. Viral metagenomics continues to offer tantalising evidence of putative mechanisms for viral piracy, but even the most advanced machine learning approaches are limited by comparison to existing model systems. Thus, if we are to better understand the impact of viral metabolic hijacking on global biogeochemical cycles, advances in computational methods must be matched with recent efforts to improve the culturing important marine taxa [108, 109] and their associated viruses, followed by in vitro determination of mechanism and impacts on host and viral fitness. Our efforts will be repaid in full as data is fed back into computational approaches to facilitate the accurate translation of experimentally observed phenotypic changes into impacts on global biogeochemical cycling in our current models.
